# White Emissions Containing Room Temperature Phosphorescence from Different Excited States of a D–*π*–A Molecule Depending on the Aggregate States

**DOI:** 10.1002/advs.202104539

**Published:** 2021-12-23

**Authors:** Mingxu Du, Yuhao Shi, Qi Zhou, Zheng Yin, Liangliang Chen, Yilin Shu, Guang‐Yan Sun, Guanxin Zhang, Qian Peng, Deqing Zhang

**Affiliations:** ^1^ Beijing National Laboratory for Molecular Sciences CAS Key Laboratory of Organic Solids Institute of Chemistry Chinese Academy of Sciences Beijing 100190 P. R. China; ^2^ University of Chinese Academy of Sciences Beijing 100049 P. R. China; ^3^ Department of Chemistry Yanbian University Jilin 133002 China

**Keywords:** aggregate state, room temperature phosphorescence, triplet state, white emission

## Abstract

Development of pure organic molecular materials with room temperature phosphorescence (RTP) and their applications for white emitters have received significant attentions recently. Herein, a D–*π*–A molecule (DMACPPY) which can realize white emitting under ambient conditions both in the crystal state and the doped‐film state by combining RTP with two fluorescent emissions is reported. The white emission from the crystalline sample of DMACPPY consists fluorescence from S_2_ (the second excited singlet state) and S_1_ (the first excited singlet state) along with RTP from T_1_ (the first excited triplet state), namely, SST‐type white light. While, the white emission from the poly methyl methacrylate (PMMA) film doped with DMACPPY contains fluorescences from S_2_ and S_1_, and RTP from T_2_ (the second excited triplet state) rather than T_1_ (STS type). DMACPPY cannot exhibit white spectrum within alternative crystalline state since inferior RTP intensity despite similar ternary emissions. The results demonstrate that the emissive properties for excited states of DMACPPY can be tuned by changing the aggregate state from crystalline to dispersion state in PMMA film. This new RTP emitter fulfills the talent for white emitting and achieves dual‐mode white emissions, invisibly, expands the application range for pure organic and heavy atom‐free RTP materials.

## Graphical abstract

A D–*π*–A molecule (DMACPPY) showing white emissions both in one crystalline state and the doped‐film state by combining room temperature phosphoresce with two fluorescent emissions is reported. The results demonstrate that the emissive properties for excited states of DMACPPY can be tuned by changing the aggregate state from crystalline to dispersion state in poly methyl methacrylate film.

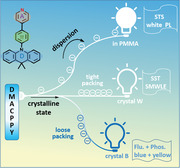

## 1 Introduction

Pure organic molecules with room temperature phosphorescence (RTP) have attracted widespread attentions in recent years due to a variety of potential applications in biological area,^[^ ^1^ ^]^ sensor,^[^ ^2^ ^]^ photoelectricity,^[^ ^3^, ^4^ ^]^ and data encryption.^[^ ^5^, ^6^ ^]^ To boost the radiation for triplet excitons, heavy atoms (such as Br and I),^[^ ^3^, ^7^, ^8^ ^]^ carbonyl groups^[^ ^9^, ^10^ ^]^ or sulfone groups,^[^ ^11^ ^]^ and donor–acceptor (D–A or D–*π*–A) structure^[^ ^2^, ^9^, ^12^, ^13^ ^]^ are anchored on the skeleton of molecular to promote spin‐orbit coupling (SOC) effect and afford efficient intersystem crossing (ISC). Meanwhile, strategies such as host–guest interactions,^[^ ^8^, ^14^, ^15^ ^]^ crystallization,^[^ ^7^, ^12^, ^16^ ^]^ polymer matrix assisting,^[^ ^2^, ^17^, ^18^ ^]^ and so on, are used to rigidify molecular configuration and thus suppress the nonradiative dissipation, including vibrational relaxation and exogenous quenching. To date, the majority of reported RTP materials exhibit yellow or green phosphorescence from the first excited triplet state T_1._^[^ ^7^, ^19^ ^]^ Combined with complementary blue fluorescence, such dual‐emission materials serve as a promising candidate for single molecule white light emitters (SMWLEs),^[^ ^9^, ^20^ ^]^ but suffer from dim phosphorescence intensity.^[^ ^20a^ ^]^ Compared to these white‐light based on multi‐emitter (two complementary colors: blue and yellow, or three primary colors: blue, red, and green),^[^ ^21^ ^]^ SMWLEs exhibit superior performances including no phase separation and color‐aging, simplified fabrication, and stability.^[^ ^22^ ^]^ However, the search of SMWLEs based on pure organic RTP materials remains in its infancy.^[^ ^13^, ^20^ ^]^ An enormous amount of research effort goes into the development of novel and efficient RTP materials to obtain balanced white spectrum,^[^ ^13^, ^20^, ^21^, ^23^ ^]^ however, ordinary or already existing complementary fluorescence‐phosphorescence dual emission materials provide a possible design for white light emission and need to be systematically examined for stimulating the white light‐emitting potential.^[^ ^9^, ^20^ ^]^ Furthermore, RTP‐active white light depends on T_1_ in large part,^[^ ^7^, ^19^ ^]^ and high‐lying triplet state (T*_n_*) participative white emission is minority and enslaved to the precise manipulation on the energy levels of different excited states.^[^ ^20^, ^23^ ^]^

**Figure 1:**
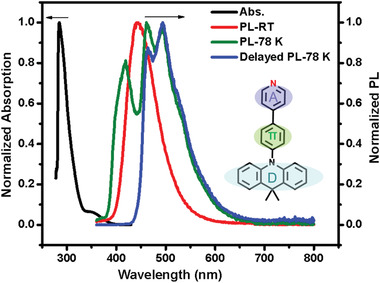
The molecular structure of DMACPPY, UV–vis absorption, emission, and delayed spectra of DMACPPY (50 µm) in toluene solution at 298 K (RT) and 78 K; the gate‐controlled delay time was 0.1 ms.

Here, by a rational molecular design, a molecule featuring with fluorescence‐phosphorescence ternary emissions in the solid state, DMACPPY, is reported (**Figure** **1**). Twisted D–*π*–A structure combined with n–π* transition endows it with tunable excited states and enriched photophysical properties. The results show that changing the configuration of DMACPPY can obtain a loose‐packing or a tight‐packing crystal morphology. The tight‐packing crystal achieves single‐molecule white emission with the Commission Internationale de l’Éclairage (CIE) 1931 coordinates of (0.27,0.26), which is composed of blue and green fluorescence along with yellow RTP. Moreover, white light consisting of ternary emissions can also be obtained by dispersing molecules of DMACPPY into poly methyl methacrylate (PMMA) thin film with a photoluminescence quantum yield (PLQY) of 40.2% and CIE coordinate of (0.26,0.30). Theoretical and experimental investigations reveal that the single‐molecule white emission from the crystalline sample is originated from high‐lying singlet state S_2_, conventional S_1_(the first excited singlet state) and T_1_ (SST‐type white light), while the white light of the doped PMMA thin film is from excited states of S_2_, T_2_ (the second excited triplet state rather than T_1_), and S_1_ (STS‐type white light). This work demonstrates that by tuning the aggregate state from crystalline to the doped thin film, the emissive properties of DMACPPY (showing both fluorescence and RTP) can be tuned to yield dual‐mode white light.

## 2 Results and Discussions

The compound DMACPPY contains 9,9‐dimethyl‐9,10‐dihydroacridine (DMAC), which is a common electron‐donating group (D)^[^ ^11^, ^24^ ^]^ and 4‐phenylpyridine (PPY), which plays the roles of *π*–bridge and weak acceptor (A) group (Figure 1). Meanwhile, the nitrogen atom on DMAC may implement the hybrid n–π* and *π*–π* transitions^[^ ^23^, ^25^ ^]^ to provide SOC effect. Twisted D–*π*–A structure coordinated with weak pyridine acceptor segment may bring about manifold locally excited (LE), charge‐transfer (CT), or mixed LE and CT processes resulting in multiple excited states. The synthesis and characterization of DMACPPY are provided in Experimental Section or Supporting Information. The chemical structure and purity were confirmed by NMR, mass spectra, and elemental analysis (see Figures S1 and S2, Supporting Information).

As shown in Figure 1, the toluene solution of DMACPPY shows relatively intense absorption (black line) in the range of 280–330 nm, which is mainly assigned as LE process based on *π*‐acceptor segment (PPY) according to theoretical calculations, see Figure S3, Supporting Information. In addition, a relatively weak absorption around 350 nm was detected and this weak absorption is attributed to the CT transition from DMAC to PPY segments, mixed with LE process from the DMAC segment. The PL spectrum of the toluene solution (red line, Figure 1) is structureless at room temperature (RT), while a hypochromatic‐shift (from 442 to 420 nm) and finely structured PL was observed at 78 K (green line) due to restricted molecular vibration relaxation at low temperature.^[^ ^26^ ^]^ Compared to delayed PL at 78 K (blue line), the structured emissions at 465 and 495 nm (green line) can be attributed to the phosphorescence of DMACPPY. The phosphorescent intensity is enhanced at 78 K to overwhelm that of the fluorescence around 420 nm, implying that DMACPPY is a promising phosphor. Referring to the solvatochromism PL spectra (Figure S4, Supporting Information), the fluorescence of DMACPPY exhibits distinct CT characteristic in polar solvent and the ISC rate is expected to be strongly accelerated consequently according to reported studies.^[^ ^2^, ^9^, ^12^, ^13^ ^]^ As shown in **Table** **1**, from toluene to DCM (dichloromethane) solutions, the fluorescence PLQY decreased from 28.7% to 24.2% and the corresponding lifetime was lengthened from 4.43 ns to 11.78 ns. In addition, the lifetime and PLQY of DMACPPY in diethyl ether is also detected as 4.81 ns and 25.1%, respectively. The results reveal that CT properties become more distinct by increasing the polarity of solvents, leading to the decrease of PLQY and extension of emission lifetime.

**Table 1:** Photophysical data for solutions, crystals of DMACPPY, and the DMACPPY doped PMMA film

	*λ*_abs_ [nm]	*λ*_F_ [nm]	*λ*_P_ [nm]	*t*_F_ [ns]	*t*_p_ [ms]	*Φ*_F_ [%]	*Φ*_P_ [%]
In toluene	285 (294), 350 (343)	443	465, 495^c)^	4.43	–	28.7	–
In DCM	253, 280, 350	512	–	11.78	–	24.2	–
Crystal B	293, 350	402	567	3	118	6.18	1.07^d)^
Crystal W	295, 350	410 (392)	567 (556)	3.4	204	6.34	2.85^d)^
In PMMA	258, 270, 287	430 (407)^a)^, 540 (528)^b)^	500 (476)	4.3^a)^, 17.2^b)^	0.7	40.2	–

^a)^ Peak at short‐wavelength region

^b)^ Peak at long‐wavelength region

^c)^ Measured at 78 K

^d)^ Integration from 500 to 800 nm. The values in brackets are based on theoretical calculations.

**Figure 2:**
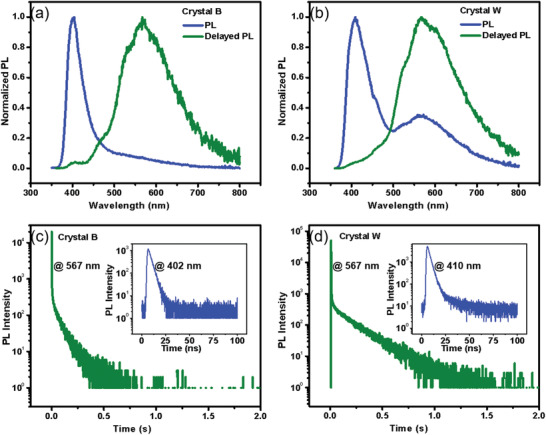
Photophysical properties for two crystalline polymorphs of DMACPPY aunder ambient conditions: a,b) PL and delayed PL spectra of crystal B and crystal W; the gate‐controlled delay time was 0.1 ms; c) PL decay curves of crystal B measured at 567 nm for long‐lived emission (green line) and at 402 nm for short‐lived emission (blue line); d) PL decay curves of crystal W measured at 567 nm for long‐lived emission (green line) and at 410 nm for short‐lived emission (blue line).

**Figure 3:**
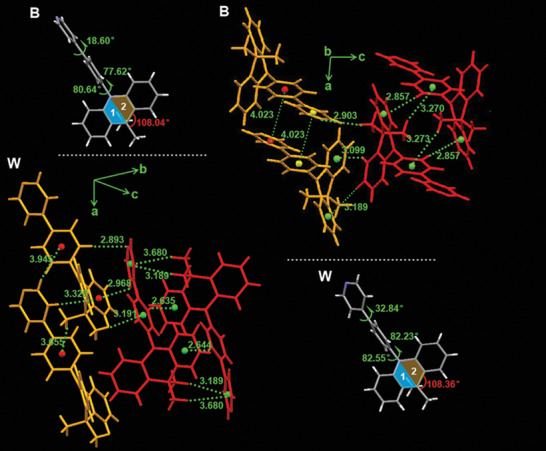
The molecular configurations and intermolecular interactions for crystal B and crystal W.

Two crystalline polymorphs of DMACPPY, referred to as crystal B and crystal W, were obtained by crystallization of DMACPPY from the mixture of DCM and acetonitrile and by diffusing n‐hexane vapor into the DCM solution of DMACPPY, respectively. **Figure** **2** shows the PL and delayed PL spectra at a gate‐controlled delay time of 0.1 ms as well as the respective decay curves for crystal B and crystal W. (**Figure** **3** and Table S1, Supporting Information shows detailed data of two crystal structures). Crystal B shows blue fluorescence at 402 nm and corresponding lifetime was determined to be 3 ns according to the decay curve (blue line in Figure 2c; see Table 1 and Figure S5, Supporting Information). In addition, the delayed PL reveals the existence of a yellow RTP emission in the region from 500 to 800 nm, and the corresponding emission peak and life time were 567 nm and 118 ms (Figure 2a,c and Table 1), respectively. Although crystal B possesses preferable complementary colors (blue fluorescence and yellow phosphorescence), it suffers from inferior phosphorescence quantum yield (*Φ*_P_ = 1.07%, see Table 1) and fails to obtain the balanced white spectrum.

**Figure 4:**
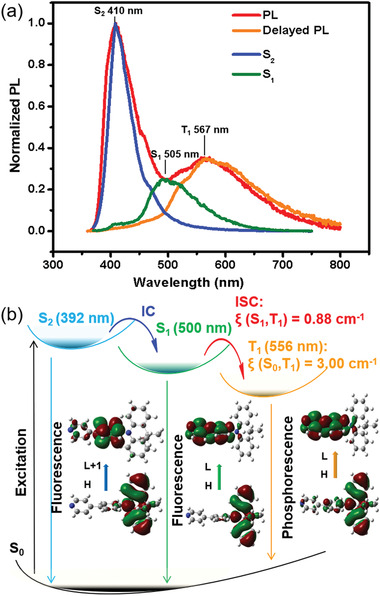
a) The steady PL, delayed PL (gate‐controlled delay time is 0.1 ms), and the emission spectra from the respective excited states of for crystal W; b) the schematic Jablonski diagram with spin‐orbit couplings (*ξ*) and transition properties of the S_2_, S_1_, and T_1_ states for DMACPPY within crystal W.

In comparison, crystal W exhibits single‐molecule white emission composed of ternary emission (**Figure** **4**). The blue fluorescence at 410 nm maintains approximate lifetime to that of crystal B (3.4 ns, see Table 1) on the basis of the decay curve (blue line in Figure 2d). The emission at 567 nm exhibits a lifetime of 204 ms (Figure 2d and Table 1). Such long‐lived emission is undoubtedly RTP in nature. Moreover, the delayed PL spectrum shown in Figure 2b is well overlapped with the emission peak at 567 nm. By comparing crystal B with crystal W, the later shows enhanced RTP with longer lifetime and higher phosphorescence quantum yield of 2.85% based on the integration from 500 to 800 nm. Interestingly, another weak fluorescence around 500 nm was detected by the time‐resolved PL spectra (Figure S6, Supporting Information), for crystal W, the lifetime of this faint green fluorescence with the emission peak at 505 nm was measured to be 6.31 ns. As a consequence, DMACPPY within crystal W shows a ternary‐emission white light with the corresponding CIE coordinates of (0.27,0.26) (see Figure 4a; Figure S5, Supporting Information), which is composed of strong blue fluorescence (≈410 nm), weak green fluorescence (≈505 nm), and the enhanced yellow RTP (≈567 nm). And other photophysical details for crystal B and crystal W are summarized in Table 1.

To further verify the origin of the multiple emissions, the temperature effect on the PL of both crystal B and W was investigated. As shown in Figure S7c,g, Supporting Information, there is hardly any variation in lifetime for two crystals at short wavelength with a decrease in temperature from 230 K to 80 K, indicating that prompt fluorescence dominants the radiation at short wavelength region. According to Figure S7a,e, Supporting Information, the PL intensities at long wavelength region, along with distinct hypochromatic‐shifts, increase by one and sevenfold approximately for crystal B and crystal W, respectively. Simultaneously, lifetimes increase obviously with decreasing the temperature from 230 K to 80 K (Figure S7d,h, Supporting Information). Taking together the results of the delayed PL (Figure S7b,f, Supporting Information) and steady PL at different temperatures, it can be concluded that the phosphorescence dominates long wavelength emission for crystal W and crystal B.

To further understand the RTP phenomenon of two crystals, the single crystal analysis was conducted. Figure 3 and Table S2 shows the molecular configurations of DMACPPY and their intermolecular interactions within crystal B and crystal W, respectively. The molecules of DMACPPY in crystal B and crystal W bear similar dihedral angles between the DMAC (D) and benzene ring (*π*), but the dihedral angle between the pyridine group (A) and benzene ring is smaller for the DMACPPY molecule in crystal B than that in crystal W. This implies that relatively efficient intramolecular charge transfer for DMACPPY molecule within crystal B. According to Figure 3 and Figure S8, Supporting Information, the adjacent molecules within crystal W are interacted with the following short interatomic contacts: C–H···*π* (2.635–3.945 Å) and C–H···N (2.893 Å). The intermolecular interactions also exist in crystal B: C–H···*π* (2.857–3.273 Å) and C–H···N (2.903 Å). In comparison, the neighboring molecules within crystal W are associated with more intermolecular interactions than those within crystal B. For example, red labeled molecule couple can achieve a sixfold C–H···*π* interactions within crystal W yet a quadruple C–H···*π* interactions within crystal B (Figure 3). Consequently, such rigid intermolecular packing may contribute to the enhanced RTP feature observed for crystal W. As it will be discussed below, by changing molecular configuration and intermolecular interactions, emissions from three excited states, especially the enhanced RTP are obtained and the white‐emitting potential of DMACPPY is activated within crystal W.

In light of the multiple emissions of crystal W, the theoretical calculations were performed to disclose the intrinsic origins, and the computational details are given in Supporting Information with the computational model in Figure S9, Supporting Information. As shown in Figure 4, Figure S10, Tables S3, S4, Supporting Information, the ternary emission (blue, green, and yellow) originates from the second, first excited singlet states S_2_, S_1_, and the first excited triplet state T_1_. More importantly, the calculated emission maxima are 392, 500, and 556 nm from S_2_, S_1_, and T_1_, respectively, which are well matched with the emission maxima 410, 505, and 567 nm measured experimentally (Figure 4 and Table 1). The emissive high‐energy S_2_ state (exception to Kasha's rule) mainly origins from the transition from HOMO (the highest occupied molecular orbital) to LUMO+1 (the second lowest unoccupied molecular orbital), which is a short‐range CT from donor moiety (DMAC) to adjacent *π* moiety (benzene) (see Figure 4b). While the S_1_ state is attributed to the CT from DMAC moiety (HOMO) to the whole phenylpyridine moieties (LUMO). The radiative rate constants of the S_1_ and S_2_ states are calculated to be 1.36 × 10^6^ and 5.62 × 10^5^ s^−1^, respectively (Table S4, Supporting Information). The ratio of the S_1_→S_2_ reverse internal conversion (rIC) to the S_2_→S_1_ internal conversion (IC) is large enough with a value of 0.005 that the population of the S_2_ is sufficient for the anti‐Kasha's emission from the S_2_ state as well as the normal emission from the S_1_ state. The T_1_ emission relies on the mixed long‐range CT (from DMAC to PPY moiety) and partially LE on the PPY moiety. Prominent SOCs *ξ* (S_2_, T_1_) and *ξ* (S_1_, T_1_) and large reorganization energy from T_2_ to T_1_ facilitate the harvest of T_1_, and the small reorganization energy suppresses the nonradiative decay of T_1_, leading to the enhanced RTP in crystal W.

**Figure 5:**
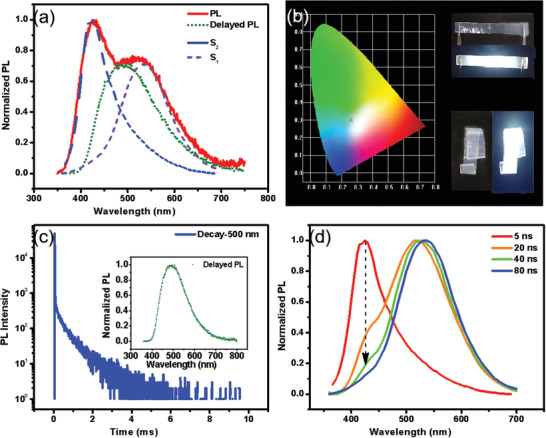
Photophysical properties for the doped PMMA film: a) The steady PL, delayed PL (gate‐controlled delay time is 0.1 ms), and the emission spectra from S_2_ and S_1_, respectively; b) *left*: CIE coordinate for 0.5% doped PMMA film, *right*: photographs for flexible white‐light illumination under daylight and 365 nm, respectively; c) delayed PL for 0.5% doped film (green dot) and the decay curve for *λ*_em_ = 500 nm; d) time‐resolved PL spectra for 0.5% doped film.

We also investigated the emission property of the DMACPPY doped‐PMMA film, aiming to improve the PL efficiency. The preparation of the doped thin film is provided in the Experimental Section. It is known that PMMA shows the excellent film‐forming ability and thus is widely used as a media to encapsulate and disperse organic phosphors.^[^ ^2^, ^17^, ^18^ ^]^ To our delight, the DMACPPY doped film (w/w for DMACPPY/PMMA = 0.5/100) shows emissions around 430, 500, and 540 nm with a white CIE of (0.26,0.30) as shown in **Figure** **5**. The delayed PL spectrum reveals the existence of persistent emission around 500 nm with a lifetime of 0.7 ms (Figure 5c). As it will be discussed below, this can be assigned as RTP from the radiative decay of the high‐lying triplet state in agreement with its higher energy than the emission around 540 nm. Time‐resolved PL spectra were performed to split the multiple emissions. As shown in Figure 5d, after delayed at 5 ns, PL spectrum mainly presented the blue short‐lived emission around 430 nm; after further delayed at 80 ns, a yellow emission around 540 nm emerged and the blue emission around 430 nm disappeared feckly. In addition, temperature‐independent decay curves (Figure S11a,b, Supporting Information) indicate the emissions around 430 and 540 nm in steady PL for doped‐PMMA film are attributed to prompt fluorescence with respective lifetimes of 7 and 17 ns. However, the emission band centered at 500 nm exhibits obvious features of triplet excitons in the temperature‐dependent experiments (Figure S11c–e, Supporting Information); the lifetime was prolonged obviously. To conclude, the white emission from the doped‐PMMA film is composed of two fluorescent emissions around 430 and 540 nm and the RTP around 500 nm. Molecular dynamics simulation (Figure S12, Supporting Information) and quantum chemistry calculations were performed to disclose the origins of the two fluorescence emissions and one RTP emission for the doped‐PMMA film. As shown in Table 1, Figure 5a, Tables S3, S4, Figure S13, Supporting Information, the high‐lying S_2_ and T_2_ (rather than T_1_), along with the conventional S_1_ excited states take the responsibility for the white emission of DMACPPY doped film. The calculated emission maxima for the ternary emissions (407 nm from S_2_, 476 nm from T_2_, and 528 nm from S_1_) approximately approach the respective experimental data: 430, 500, and 540 nm (Table 1). Furthermore, the transition characters of the two singlet states are similar with those in crystal W, resulting in similar emission property with significantly comparable radiative rate constants (2.41 × 10^5^ s^−1^ for the S_2_ state and 8.26 × 10^6^ s^−1^ for the S_1_ state). Comparing with the triplets of crystal W in Figure S10, Supporting Information, the triplets for the doped thin film were altered largely (see Figures S12 and S13). The CT character in T_1_ becomes more obvious owing to the increase of the HOMO→LUMO component (CT from DMAC to phenylpyridine), which can reduce the energy of the T_1_ state. On the contrary, the LE character in T_2_ gets much more pronounced comparing with that in crystal W. The disparity between T_1_ and T_2_ in the doped thin film results in completely different phosphorescence that is different from that in crystal W. The larger SOC *ξ* (S_2_, T_2_) than *ξ* (S_2_, T_1_) and much smaller reorganization energy from T_2_ to T_1_ both are conducive to the population of T_2_ rather than T_1_. Moreover, the reorganization energy (*λ*) between T_2_ and S_0_ (ground state) is so far smaller than that between T_1_ and S_0_, which suggests much faster nonradiative decay of T_1_ than that of T_2._ Thus, the theoretical calculations agree with the observation of the anti‐Kasha's phosphorescence of T_2_. As a result, a STS‐type white light‐emission is achieved by dispersing DMACPPY into the PMMA film. The doped PMMA film shows a high quantum yield of 40.2% that is much higher than those of crystal W and crystal B. Such high emission quantum yield can be attributed to the following factors: i) PMMA serves as a rigid matrix to suppress vibrational relaxation for DMACPPY, and ii) molecules of DMACPPY are dispersed in the film at a very low concentration, which profits the radiative decay. As shown in Figure S15, Supporting Information, the PL spectrum of DMACPPY doped film at a weight ratio of 0.5% is almost the same as that for the doped film at a weight ratio of 0.1% or 1.0%. Overall, by changing from crystalline state to dispersion state, the radiative transition channel for T_2_ is switched on and as a result the RTP is evidently hypochromatic‐shift by comparing with that of crystal W. In addition, the radiative transition channels for S_2_ and S_1_ maintain valid in the doped thin film. At last, an application of white‐light illumination was demonstrated with the DMACPPY‐doped PMMA film combined with polyethylene (PE) flexible substrate. As shown in Figure 5b, high quality white light emission was achieved in both stretched shape and elastic bent shape.

## 3 Conclusion

In summary, we report a new RTP emitter (DMACPPY) exhibiting white emissions from multiple excited states. Moreover, the emissive properties of DMACPPY were found to be dependent on the aggregate state. One crystalline form (crystal W) shows RTP around 567 nm and two fluorescent peaks around 410 and 505 nm, and the quantum yield and lifetime of RTP are 2.85% and 204 ms, respectively. For another crystalline form (crystal B), two fluorescences and a rather weak RTP were detected. The different emissive properties of these two crystalline forms of DMACPPY are attributed to the fact that molecules of DMACPPY in crystal W are associated with more intermolecular interactions, and as a consequence, molecular vibrations are reduced, which is favorable for RTP. Further theoretical calculations show that the white emission of crystal W, which is composed of RTP around 567 nm and two fluorescences around 410 and 505 nm, is originated from excited states of T_1_, S_2_, and S_1_, respectively. Alternatively, RTP and white emission were also observed for the DMACPPY‐doped PMMA thin film and the white emission quantum yield reached 40.2%. Interestingly, the RTP for such doped thin film comes from T_2_, while the fluorescences around 430 and 540 nm are from S_2_ and S_1_, respectively, on the basis of theoretical calculations. Finally, the DMACPPY‐doped PMMA film was utilized to demonstrate the application of white‐light illumination in combination with PE flexible substrate.

## 4 Experimental Section

### Synthesis of DMACPPY

To a toluene solution (50 mL) of DMAC (2.0 g, 9.56 mmol), were added 4‐(4‐bromophenyl)pyridine (1.48 g, 6.36 mmol), *t*BuOK (2.24 g, 20 mmol), Pd(OAc)_2_ (0.05 g, 0.22 mmol), and P(*t*Bu)_3_·HBF_4_ (0.32 g, 1.1 mmol). The resulting mixture was refluxed for 24 h under N_2_ atmosphere. After cooling to RT, the reaction mixture was poured into water and then extracted with dichloromethane (DCM). The combined organic layers were dried over anhydrous Na_2_SO_4_. After filtration and evaporation, the product was purified with silica‐gel column chromatography using DCM/methanol (100:1, v/v) as eluent, DMACPPY as a pale solid was obtained in a yield of 60%. This product was further purified by two‐time recrystallization from the mixture of DCM and methanol (v/v, 1/5). The product was further purified with temperature‐gradient sublimation under vacuum. ^1^H NMR (300 MHz, CD_2_Cl_2_, *δ*): 8.71 (d, *J* = 6.1 Hz, 2H), 7.95 (d, *J* = 8.5 Hz, 2H), 7.67 (d, *J* = 6.2 Hz, 2H), 7.48 (d, *J* = 8.4 Hz, 4H), 7.07–6.81 (m, 4H), 6.32 (d, *J* = 7.6 Hz, 2H), 1.69 (s, 6H).^13^C NMR (75 MHz, CDCl_3_, *δ*): 150.52, 147.64, 142.39, 140.85, 138.09, 132.26, 130.32, 129.64, 126.54, 125.49, 121.82, 120.93, 114.15, 36.15, 31.40. MS (MALDI‐TOF): *m*/*z* [M^+^] calculated for C_26_H_23_N_2_ 363.1855; found, 363.1853. Analytically calculated for C_26_H_22_N_2_: C 86.15, H 6.12, N 7.73; found: C 86.14, H 6.24, N 7.70.

### Preparation of DMACPPY Doped‐PMMA Film

First, 50.0 mg of PMMA (average molecular weight is 35 000) and 0.25 mg of DMACPPY were dissolved in 2 mL of DCM, and the mixed solution was stirred over 3 h at 35 ℃. 40 µL of the mixed solution was used to prepare amorphous film by drop‐coating method on quartz or PE substrate. Thin film was dried at 35 ℃ over 1 h until the solvent was evaporated.

## Conflict of Interest

The authors declare no conflict of interest.

## Supporting information

Supporting Information

## Data Availability

The data that support the findings of this study are available in the supplementary material of this article.
