# Supplementary material for: White Emissions Containing Room Temperature Phosphorescence from Different Excited States of a D–π–A Molecule Depending on the Aggregate States
Source: Adv Sci (Weinh). 2021 Dec 23;9(5):2104539. doi: 10.1002/advs.202104539 (PMC8844470; doi:10.1002/advs.202104539)
Supplement: Supplementary file 1 — Supporting Information [file ADVS-9-2104539-s001.pdf]

© 2020 Wiley-VCH GmbH

ADVANCED SCIENCE
Open Access

# Supporting Information

for *Adv. Sci.,* DOI: 10.1002/advs.202104539

# White emissions containing room temperature phosphorescence from different excited states of a D-π-A molecule depending on the aggregate states

*Mingxu Du, Yuhao Shi, Qi Zhou, Zheng Yin, Liangliang Chen, Yilin Shu, Guang-Yan Sun, Guanxin Zhang, Qian Peng*, and Deqing Zhang**

Supporting Information

**White emissions containing room temperature phosphorescence from different excited states of a D-π-A molecule depending on the aggregate states**

*Mingxu Du, Yuhao Shi, Qi Zhou, Zheng Yin, Liangliang Chen, Yilin Shu, Guang-Yan Sun, Guanxin Zhang, Qian Peng, * Deqing Zhang**

Dr. M. Du, Q. Zhou, Z. Yin, L. Chen, Y. Shu, Dr. G. Zhang, Prof. D. Zhang

Beijing National Laboratory for Molecular Sciences, CAS Key Laboratory of Organic Solids, Institute of Chemistry, Chinese Academy of Sciences, Beijing, 100190, P. R. China.
E-mails: dqzhang@iccas.ac.cn
Y. Shi, Q. Zhou, Z. Yin, L. Chen, Y. Shu, Dr. G. Zhang, Prof. Q. Peng, Prof. D. Zhang
University of Chinese Academy of Sciences, Beijing 100049, P. R. China
E-mails: qianpeng@ucas.ac.cn
Y. Shi, Prof. G. Sun
Department of Chemistry, Yanbian University, Yanji, Jilin 133002, China

Scheme S1 Synthetic route of DMACPPY.

Figure S1 and S2 NMR spectra for DMACPPY.

Figure S3 Theoretical investigations for absorptions in toluene.

Figure S4 Solvatochromism of emission spectra.

Figure S5 CIE coordinates for crystal B and crystal W.

Figure S6 Time-resolved PL spectra for crystal B and crystal W.

Figure S7 Temperature effect on the photophysical properties of two crystals.

Figure S8 Packing modes for crystal B and crystal W from different viewing angles.

Figure S9 QM/MM modeling of crystal B and crystal W

Figure S10 Calculated results of crystal W.

Figure S11 Temperature effect on the photophysical properties of the doped film

Figure S12 RMSD analysis and the dynamic simulation of the doped PMMA film

Figure S13 Calculated results of doped thin film

Figure S14 Absorption spectra for DMACPPY in different conditions:

Figure S15 The PL spectra of doped film with different doping concentration
Figure S16 Low-temperature delayed PL spectra of DMACPPY in different solvents.
Figure S17 Theoretical investigations for DMACPPY within crystal B

Table S1 Structural data of crystal B and crystal W.
Table S2 Comparison of packing model for Crystal B and Crystal W.
Table S3 and Table S4 Calculated results for crystal W and the doped PMMA film.

## Materials and Characterization techniques

*General*: Reagents were purchased from Innochem, Energy-Chemical and used without further purification, if not specified elsewhere. Solvents were dried or distilled out before being used for the synthesis. $^{1}$H NMR and $^{13}$C NMR spectra were recorded on Bruker Fourier 300 MHz spectrometer at 298 K. High-resolution mass spectrometry (HRMS) was recorded on a Bruker Solarix-XR high-resolution mass spectrometer operating in MALDI-TOF mode. Elemental analysis was conducted on a Carlo-Erba-1106 instrument. Single-crystal X-ray diffraction measurements were conducted on a Rigaku Saturn diffractometer with CCD area detector at 170 K.

*Photophysical measurements*: The absorption spectra of the solution and solid samples were recorded on HITACHI UH4150 UV−Visable spectrophotometer and SHIMADZU UV-2600, respectively. PL spectra of the solution and solid samples were recorded on Edinburgh FS5 and Edinburgh FLS980. The PLQYs of the solution and solid samples were measured on HAMAMATSU C11347 and Edinburgh FLS980 with an integrating sphere, respectively. The delayed PL spectra were measured on Edinburgh FLS980. The temperature-dependent spectra and lifetimes were measured on an Edinburgh FLS-980 equipped with a xenon laser arc lamp (Xe900), a microsecond flash lamp (uF900), and a picosecond pulsed diode laser. For the

measurement of fluorescence lifetimes, the picosecond pulsed diode laser (EPL- 310 nm) was used as excitation source. For phosphorescence lifetimes, the microsecond flash lamp was used with the excitation wavelength of 310 nm. The time-resolved PL spectra were measured by Edinburgh FLS-980 (operated in TRES mode, selected timescale is 100 ns; detector is Visible PMT; Stop condition times is 60 s) equipped with picosecond pulsed diode laser (EPL- 310 nm, selected frequency is 100 Hz). Based on the decay curves of lifetime at different wavelength values covering the whole PL spectra, by data slicing at a certain moment, the corresponding transient spectrum is obtained.

*Theoretical calculations*: The quantum mechanics /molecular mechanics (QM/MM) method was used to calculate the geometrical and electronic structures of the compound in crystalline states by adopting two-layer ONIOM model [S1] in Gaussian 16 program[S2]. The computational models were built from the X-ray crystal structures and shown in Figure S9, in which the central molecule was treated as QM region by (TD) B3LYP/6-31G(d) and the surrounding ones were handled as MM region by UFF force field. The spin-orbit couplings (SOC, $\xi$) and nonadiabatic couplings were calculated at the level of (TD) B3LYP/6-31G (d) using Q-Chem 5.3 program [S3]. The reorganization energies were calculated by the four-point method according to the adiabatic potential (AP) energy surface in the ground state and excited states.

The molecular dynamics (MD) simulation was performed to mimic DMACPPY doped film using GROMACS package[S4]. The atom types and parameters of DMACPPY and PMMA were built by general amber force field (GAFF) [S5]. The partical charges of every atom of the two molecules were produced from the electrostatic potential by using the restrained electrostatic potential (RESP) fit method.[S6-S7] The energy minimization for the system was firstly implemented to

stabilize configuration using the steepest descent algorithm, then followed by 500 ps MD simulations under the NVT (300 K) ensemble to relax the heavy atoms, and finally the 180 ns MD simulations were performed under the NPT ensemble with T = 300 K and P = 1 bar. Weak couplings to external heat and pressure baths were applied based on the velocity rescaling thermostat[S8] and Parrinello–Rahman barostat[S9] schemes, respectively. Periodic boundary conditions were applied in all directions to minimize the edge effects in a finite system. Newton's classical equations of motion were integrated at a time step of 2 fs using the classical leapfrog algorithm. From root-mean-square deviation (RMSD) analysis (Figure S12), we knew that the simulated film has reached an equilibrium, and chose the final 10 ns of MD simulations for the model for the further QM/MM computation (see Figure S12) at the same level[S10].

The radiative rate constant was calculated by using the Einstein formula of $k_{\mathrm{r}} = \frac{f\Delta E^2}{1.499}$. The nonradiative rate constant was calculated by using the thermal vibration correlation function rate theory in MOMAP program developed by Shuai and Peng groups. [S11-S13]

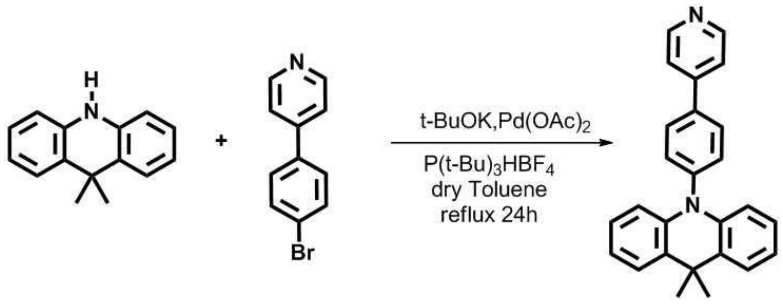


Scheme S1 Synthetic route of DMACPPY.

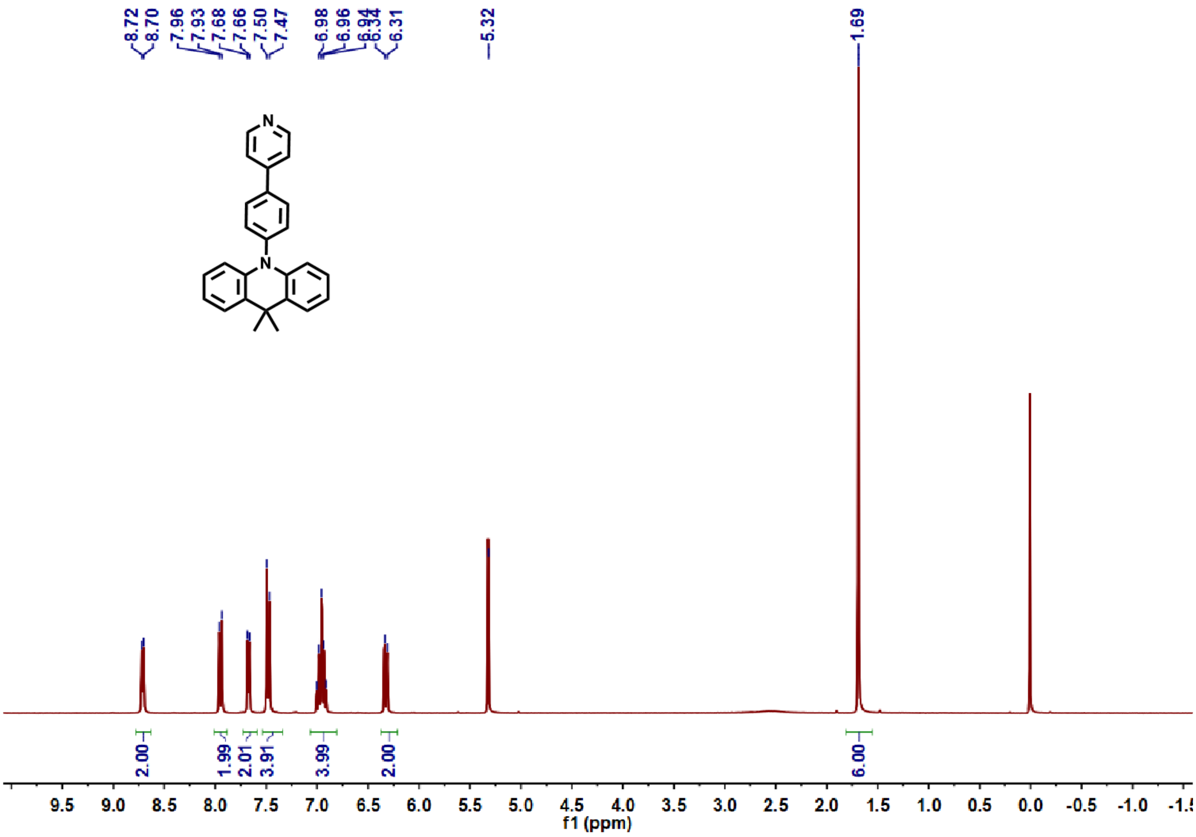


Figure S1 $^{1}H$ NMR spectrum for DMACPPY in $CD_2Cl_2$.

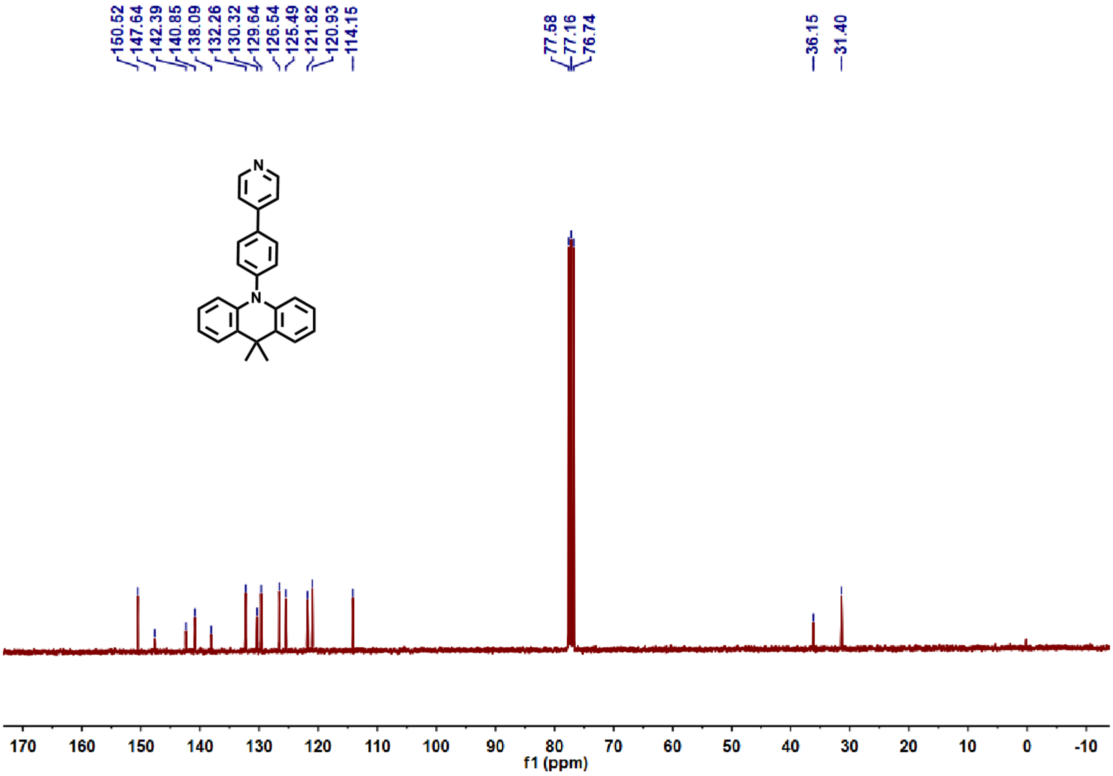


Figure S2 $^{13}C$ NMR spectrum for DMACPPY in $CDCl_3$.

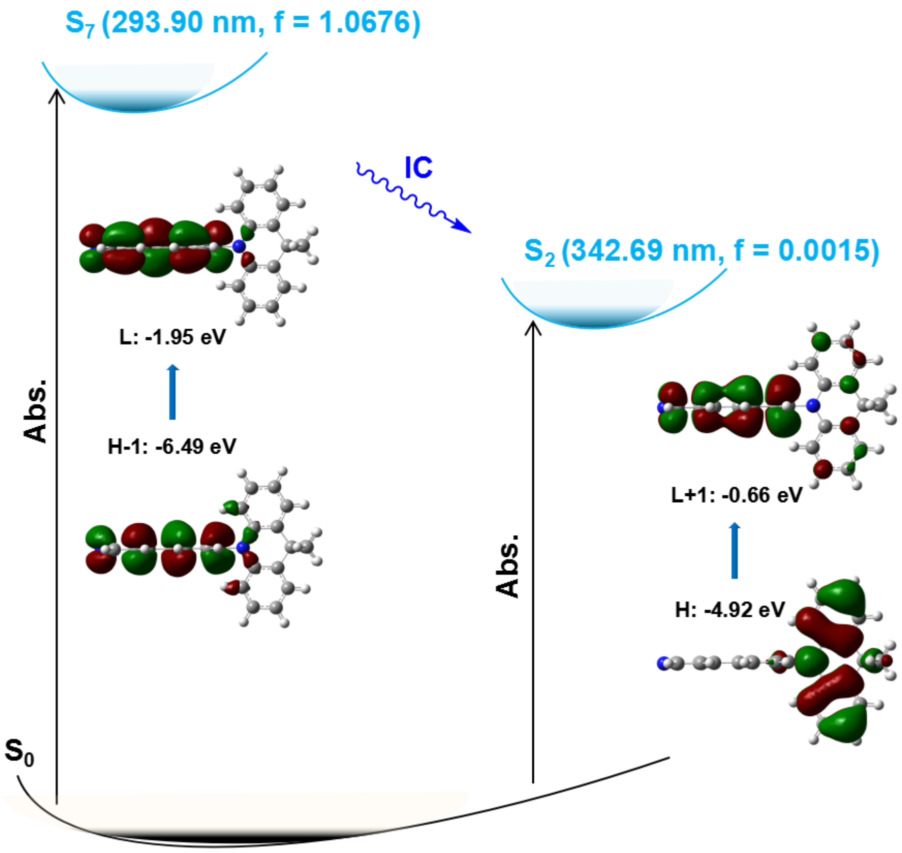


Figure S3 Theoretical investigations (absorption of $S_7$ and $S_2$) for DMACPPY in toluene: calculated absorption maximum, relevant oscillator strengths (f), transition properties of molecular orbitals and calculated energy levels for frontier molecular orbitals (FMO).

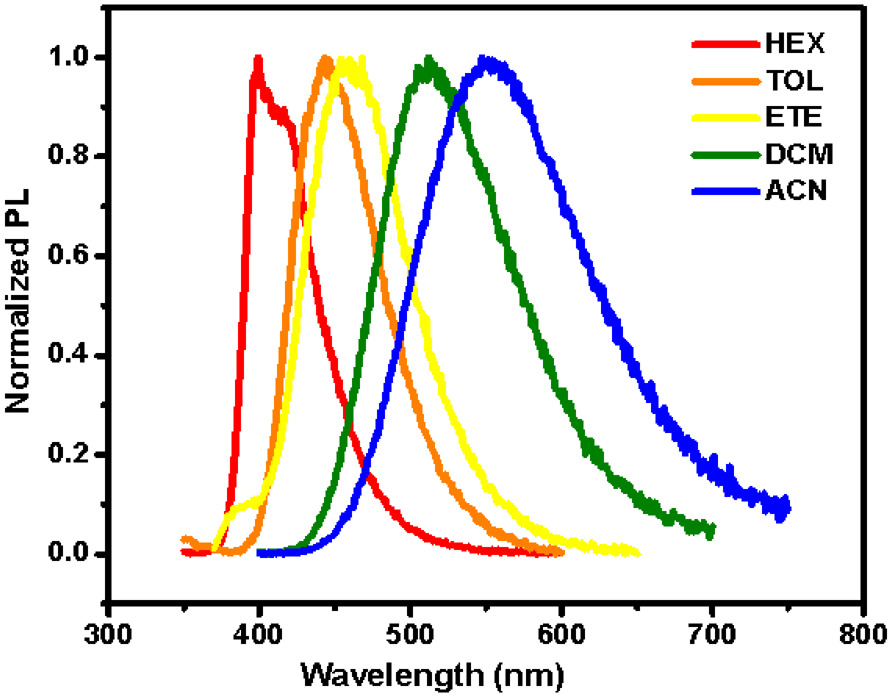


Figure S4 Solvatochromism of emission spectra of DMACPPY (50 μM) in different solvents. HEX: *n*-hexane, TOL: toluene, ETE: diethyl ether, DCM: dichloromethane, ACN: acetonitrile.

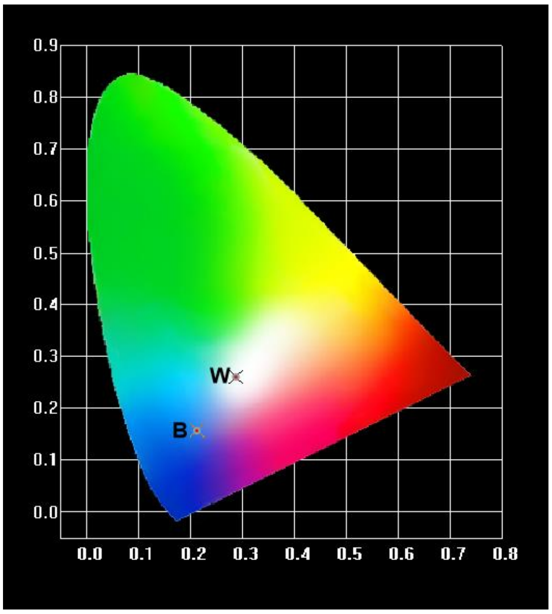


Figure S5 CIE coordinates for emissions from crystal B and crystal W.

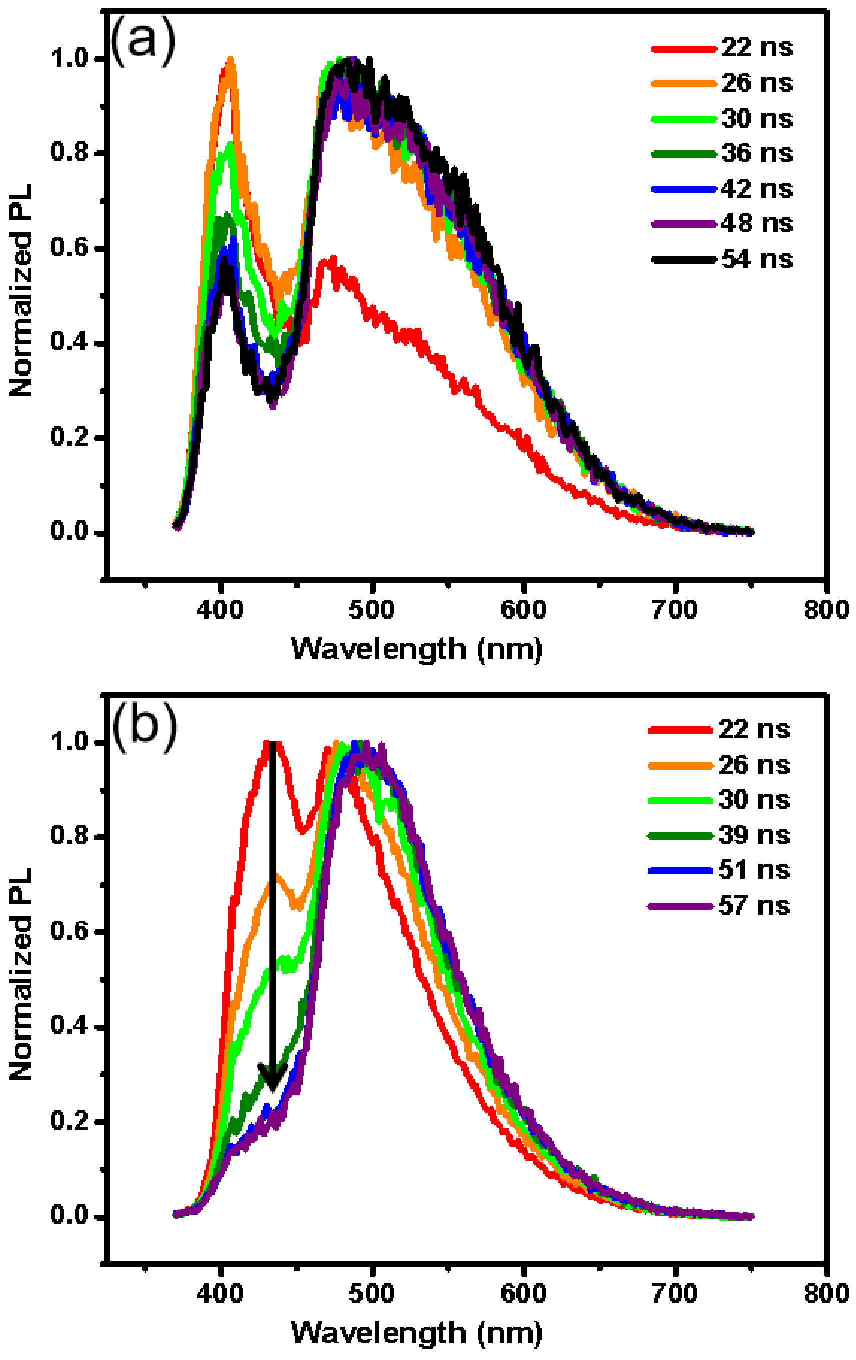


Figure S6 Time-resolved PL spectra: (a) for crystal B, (b) for crystal W.

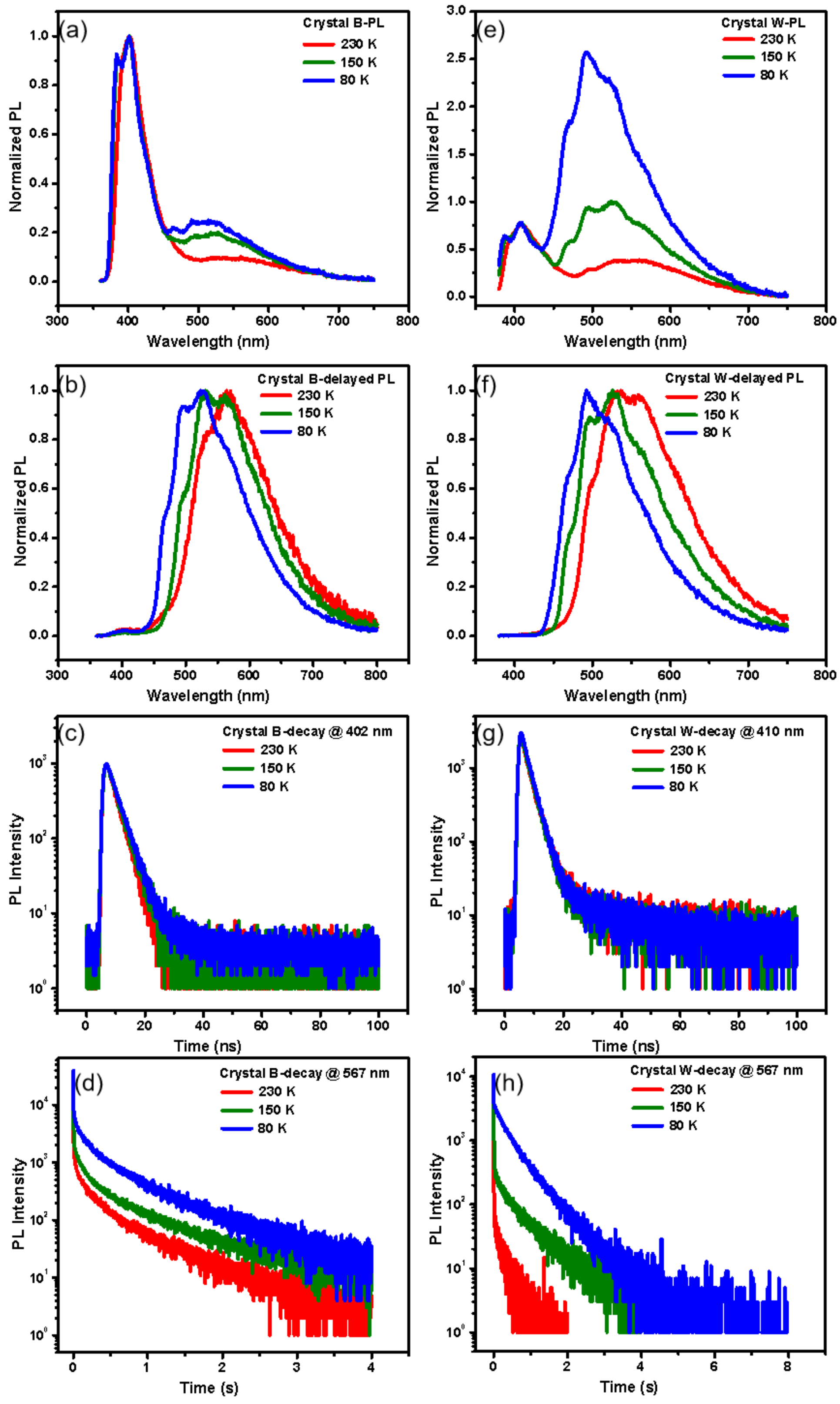


Figure S7 (a) Normalized PL spectra measured at different temperatures for crystal B; (b) Delayed PL spectra measured at different temperatures for crystal B

(gate-controlled delay time is 0.1 ms); (c) Nanosecond decay profile of crystal B measured at 402 nm; (d) Second-scale PL decay curve measured at 567 nm for crystal B; (e) Normalized PL spectra measured at different temperatures for crystal W; (f) Delayed PL spectra measured at different temperatures for crystal W (gate-controlled delay time is 0.1 ms); (g) Nanosecond decay profile of crystal W measured at 410 nm; (h) Second-scale PL decay curve measured at 567 nm for crystal W.

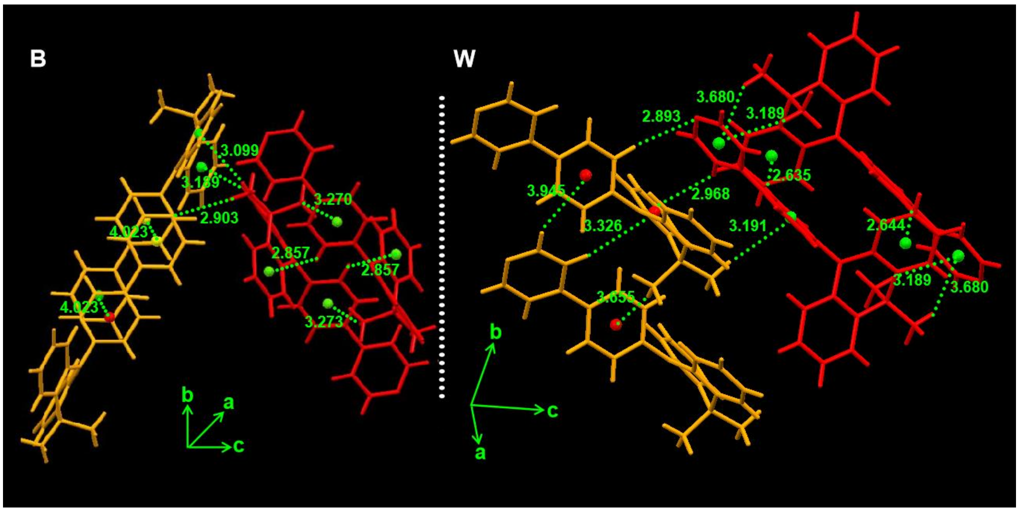


Figure S8 Intermolecular packing and interactions for crystal B and crystal W from different viewing angles.

Table S1 Structural data for crystal B and crystal W.

| DMACPPY | Crystal B | Crystal W |
|---|---|---|
| Formula | $C_{26}H_{22}N_2$ | $C_{26}H_{22}N_2$ |
| molecular weight | 362.45 | 362.45 |
| crystal system | monoclinic | monoclinic |
| space group | $P2_1/n$ | $P2_1/n$ |
| crystal size [$mm^3$] | 0.049 × 0.012 × 0.011 | 0.030 × 0.020 × 0.010 |
| a [Å] | 11.44290(10) | 8.5187(3) |
| b [Å] | 8.47910(10) | 15.0544(6) |
| c[Å] | 19.5166(2) | 15.1181(5) |
| α[$^o$] | 90 | 90 |
| β[$^o$] | 92.4390(10) | 93.649(3) |
| γ[$^o$] | 90 | 90 |
| V [Å $^3$] | 1891.89(3) | 1934.89(12) |
| Z | 4 | 4 |
| $\rho_{calc}$ [g $cm^{-3}$] | 1.273 | 1.244 |
| temp [K] | 170(2) | 170(2) |
| μ [$mm^{-1}$] | 0.570 | 0.558 |
| reflections collected | 13918 | 12757 |
| independent reflections | 3748 [$R_{int}$ = 0.0238, | 3785 [$R_{int}$ = 0.0637, |
| RF, Rw($F^2$) (all data) | $R_{sigma}$ = 0.0224] | $R_{sigma}$ = 0.0394] |
| RF, Rw($F^2$) [I > 2σ(I)] | $R_1$ = 0.0393, $wR_2$ = | $R_1$ = 0.1033, $wR_2$ = |
| GOF | 0.0988 | 0.2726 |
| CCDC | $R_1$ = 0.0366, $wR_2$ = | $R_1$ = 0.0949, |
| | 0.0967 | $wR_2$ = 0.2633 |
| | 1.030 | 1.090 |
| | 2045386 | 2045385 |

Table S2 Comparison of intermolecular interactions between Crystal B and Crystal W

| | Crystal B | Crystal W |
|---|---|---|
| Couple Y | π···π interactions (4.023 Å) | C-H···π interactions (3.655 Å, 3.326 Å, 3.945 Å) |
| Couple R | C-H···π interactions (3.270 Å, 2.857 Å, 3.273 Å) | C-H···π interactions (3.680 Å, 3.189 Å, 2.635 Å and 2.644 Å) |
| Between Couple Y and Couple R | C-H···π interactions (3.099-3.189 Å) C-H···N interactions (2.903 Å) | C-H···π interactions (3.191 Å or 2.968 Å) interactions (2.893 Å) |

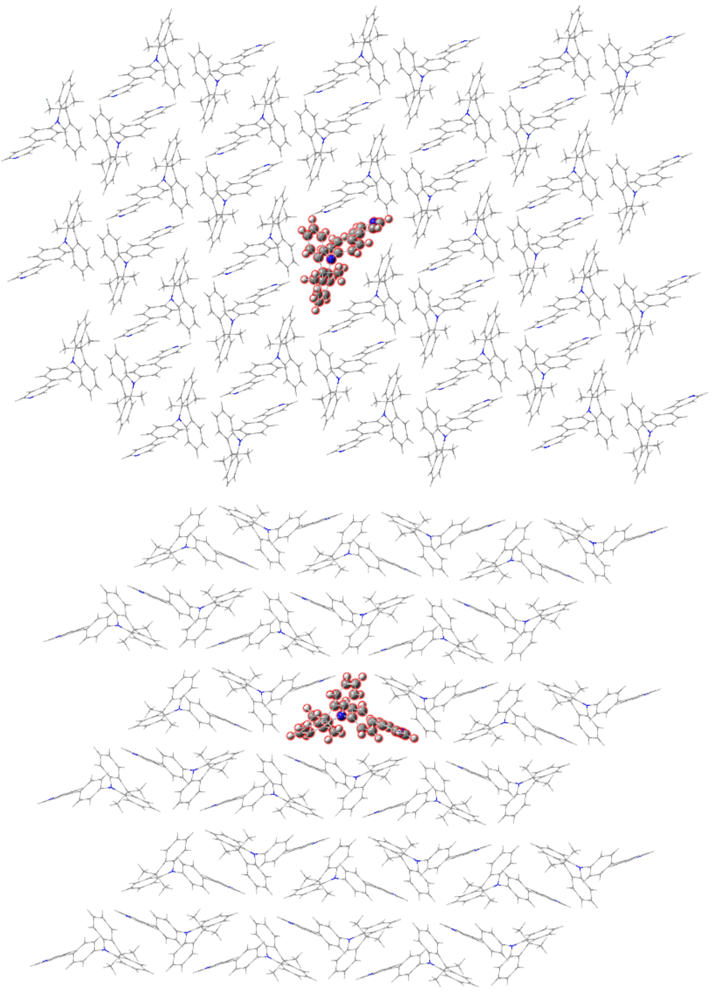

Figure S9 QM/MM modeling of DMACPPY molecule in crystal B (up) and crystal W (down). Note that the central molecule is treated using QM method and the surrounding molecules are handled by MM method.

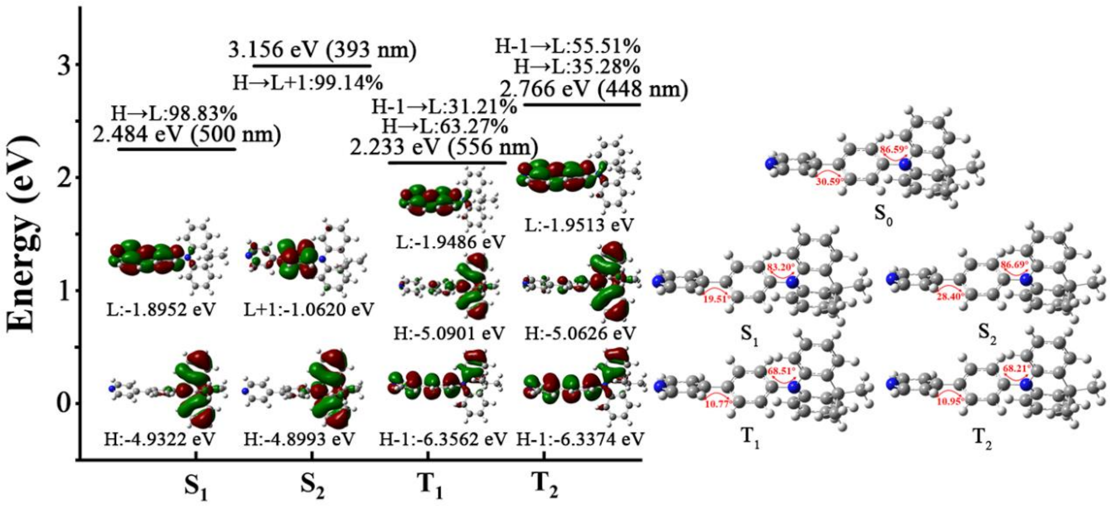


Figure S10 Calculated adiabatic excitation energies, transition properties and charge density of the involved frontier molecular orbitals based on the optimized geometries

in the $S_0$, $S_1$, $S_2$, $T_1$ and $T_2$ states for crystal W, respectively. The geometries in the $S_0$ was optimized at B3LYP/6-31G(d) level and those in the $S_1$, $S_2$, $T_1$ and $T_2$ states were optimized at TD B3LYP/6-31G(d) level. All the excitation energies were calculated at TD B3LYP/6-31G(d) level.

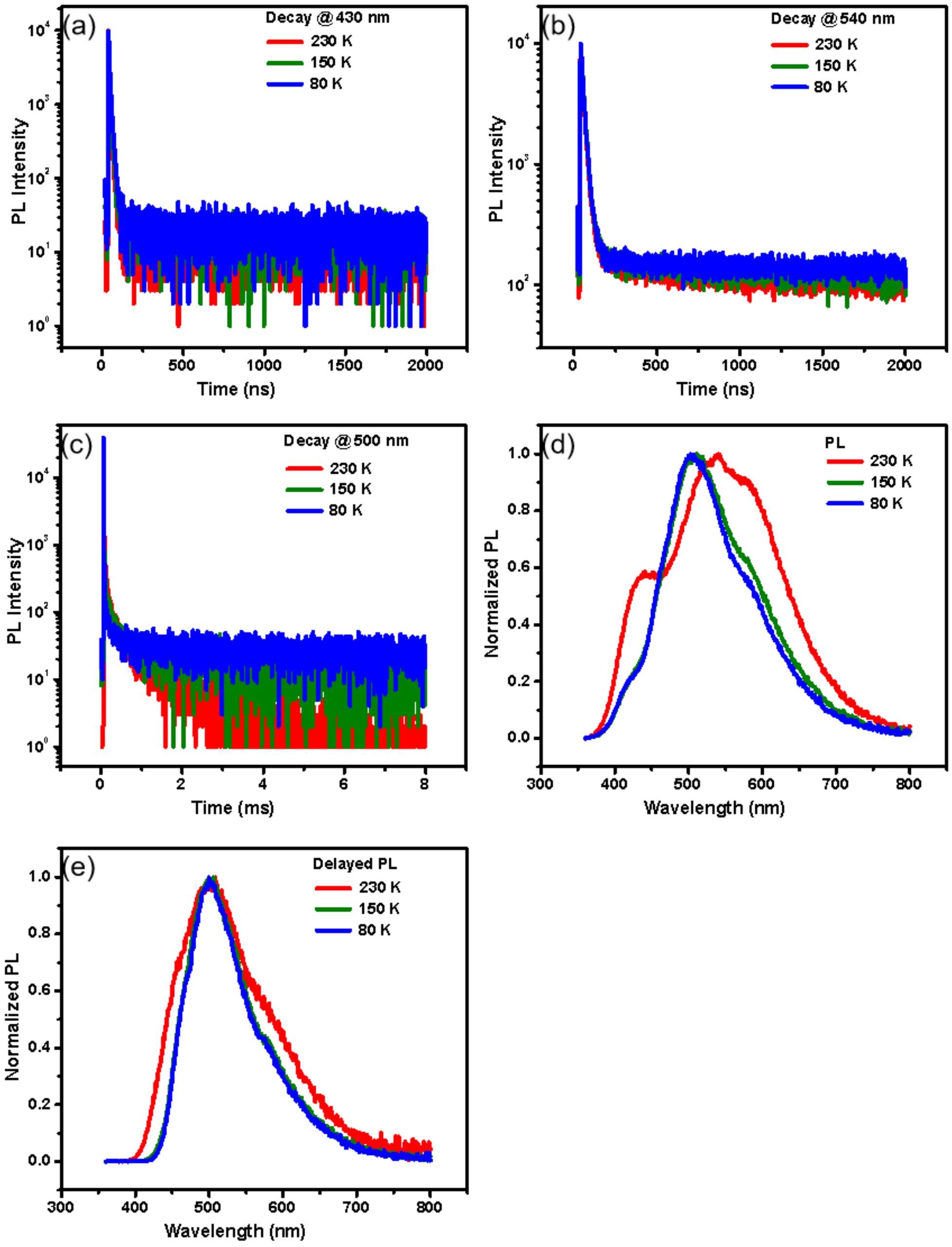


Figure S11 Temperature effect on the photophysical properties of the doped PMMA film: (a) Nanosecond decay profile measured at 430 nm; (b) Nanosecond decay profile measured at 540 nm; (c) Millisecond-scale PL decay curve measured at 500

nm; (d) PL spectra measured at different temperatures; (e) Delayed PL spectra measured at different temperatures (gate-controlled delay time is 0.1ms).

Table S3 Calculated spin-orbit couplings (ξ) ($cm^{-1}$) and reorganization energy (λ) ($cm^{-1}$) for crystal W, doped PMMA film and single-molecule model.

| | $S_1 \rightarrow S_0$ | $S_2 \rightarrow S_0$ | $T_2 \rightarrow T_1$ | $S_2 \rightarrow T_1$ | $S_2 \rightarrow T_2$ | $S_1 \rightarrow T_1$ | $T_1 \rightarrow S_0$ | | $T_2 \rightarrow S_0$ | |
|---|---|---|---|---|---|---|---|---|---|---|
| | λ | λ | λ | ξ | ξ | ξ | ξ | λ | ξ | λ |
| Crystal W | 2416.41 | 2841.96 | 7397.66 | 1.18 | 0.32 | 0.88 | 3.00 | 2430.00 | 2.16 | 937.87 |
| In PMMA | 2231.15 | 2083.73 | 1310.18 | 0.26 | 1.56 | 1.14 | 1.54 | 3244.87 | 2.09 | 1379.38 |

Table S4 Calculated energy gap (eV), oscillator strength (*f*) rate constants ($s^{-1}$) of radiative transition.

| | $\Delta E_{S1 \rightarrow S0}$ | $f_{S1 \rightarrow S0}$ | $k_{rS1 \rightarrow S0}$ | $\Delta E_{S2 \rightarrow S0}$ | $f_{S2 \rightarrow S0}$ | $k_{rS2 \rightarrow S0}$ |
|---|---|---|---|---|---|---|
| Crystal W | 2.48 | $5.1 \times 10^{-3}$ | $1.36 \times 10^{6}$ | 3.16 | $1.3 \times 10^{-3}$ | $5.62 \times 10^{5}$ |
| In PMMA | 2.35 | $3.5 \times 10^{-2}$ | $8.26 \times 10^{6}$ | 3.04 | $2.9 \times 10^{-3}$ | $2.41 \times 10^{5}$ |

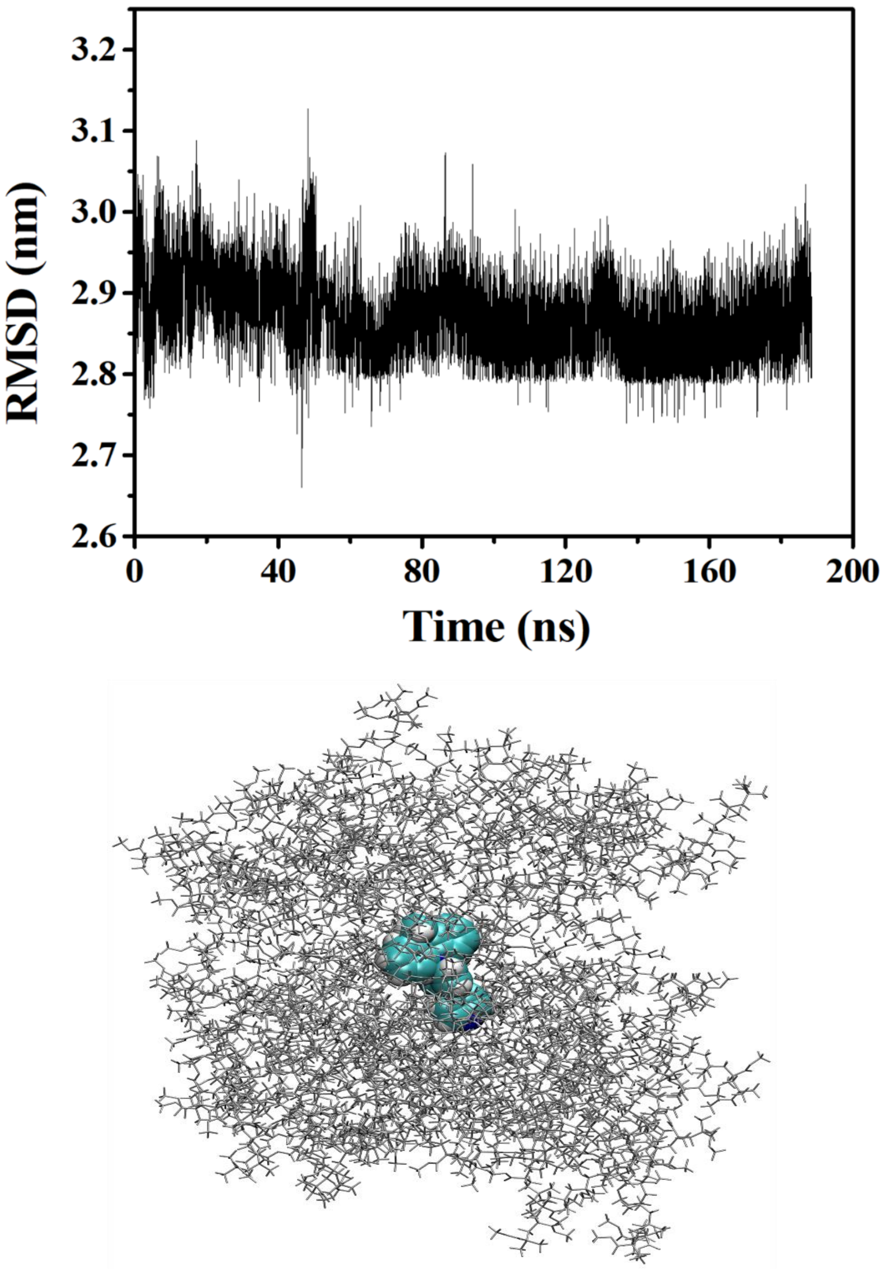


Figure S12 The root-mean-square deviation (RMSD) analysis of the doped PMMA film (*up*) during the dynamic simulation and the selected QM/MM model (*down*).

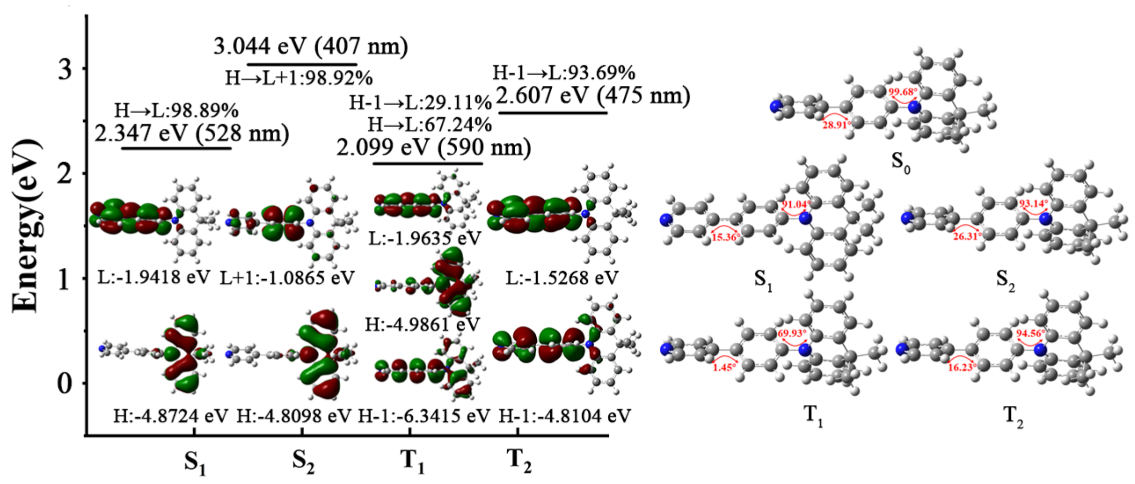


Figure S13 Calculated adiabatic excitation energies, transition properties and charge density of the involved frontier molecular orbitals based on the optimized geometries in the $S_0$, $S_1$, $S_2$, $T_1$ and $T_2$ states, respectively for the molecule in the doped thin film. The geometries in the $S_0$ is optimized at B3LYP/6-31G(d) level, those in the $S_1$, $S_2$, $T_1$ states were optimized at TD-B3LYP/6-31G(d) level while the ones in the $T_2$ were optimized at TDA-B3LYP/6-31G(d) level. All the excitation energies were calculated at TD-B3LYP/6-31G(d) level without considering the environment.

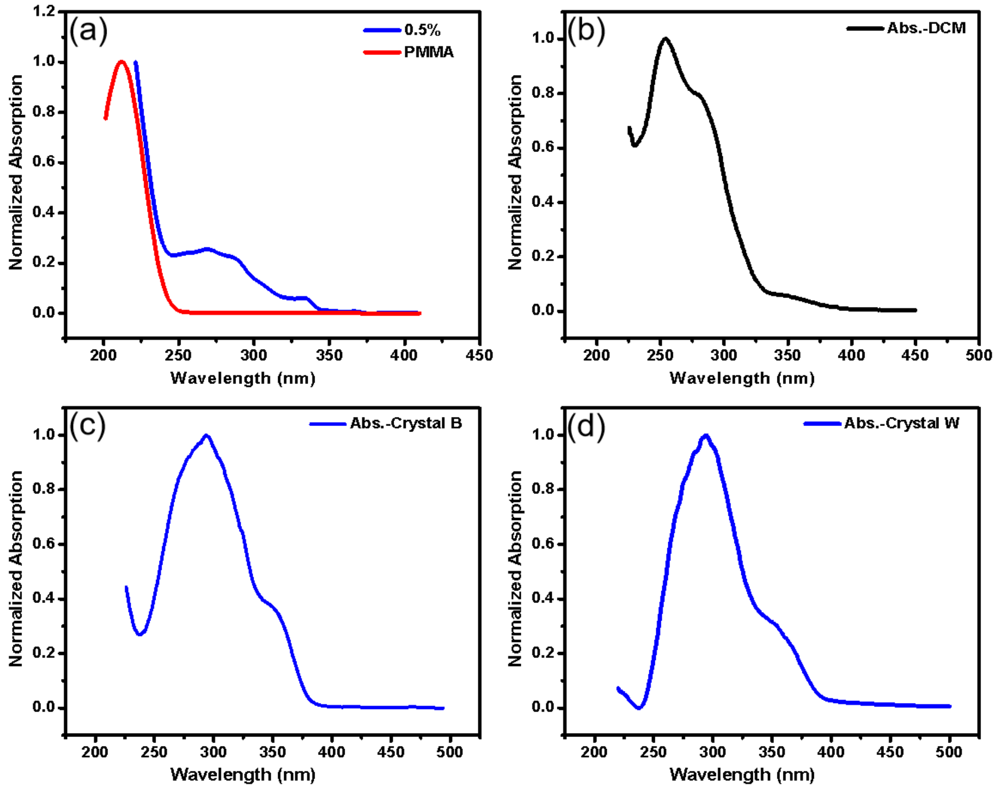


Figure S14 Absorption spectra for DMACPPY in different conditions: (a) DMACPPY doped PMMA film (0.5%) and neat PMMA film; (b) DMACPPY in DCM solution (50 μM); (c) In crystal B state; (d) In crystal W state.

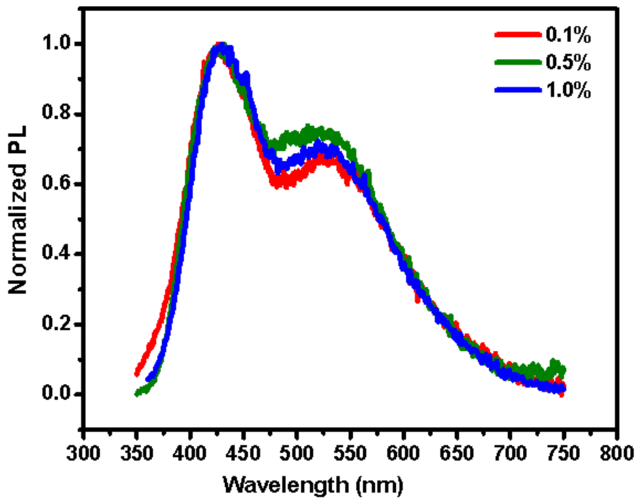


Figure S15 The PL spectra of DMACPPY doped PMMA film with different doping weight ratios (0.1%, 0.5%, 1.0%).

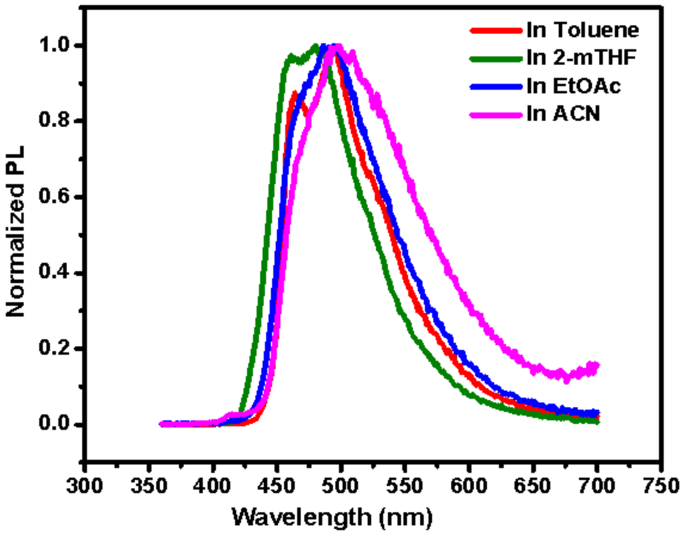


Figure S16 Low-temperature (78 K) delayed PL spectra of DMACPPY (50 μM, the gate-controlled delay time was 0.1ms) in toluene, 2-Methyltetrahydrofuran (2-mTHF), ethyl acetate (EtOAc), and acetonitrile (ACN) solution, respectively.

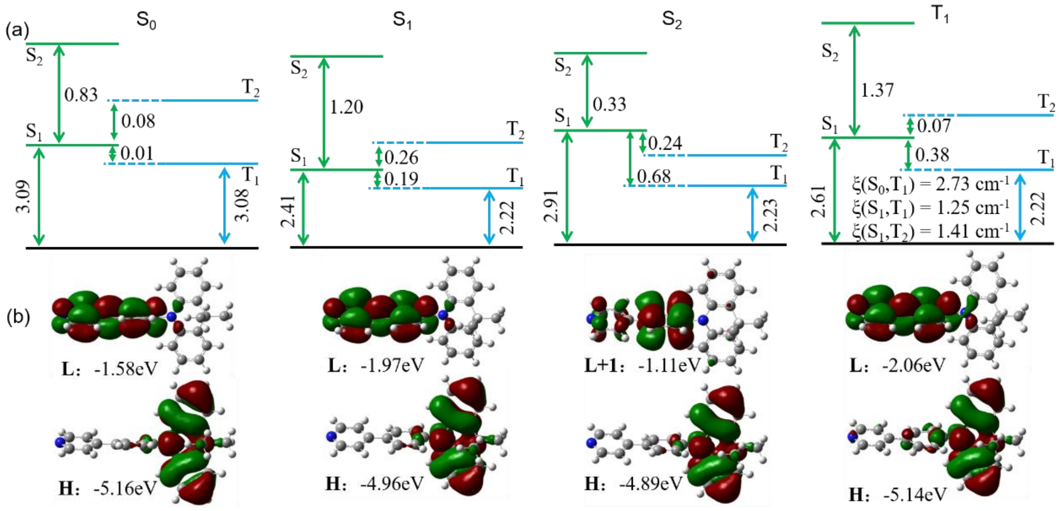


Figure S17 Theoretical investigations ($S_0$, $S_1$, $S_2$, $T_1$) for DMACPPY in crystal B: (a) The calculated energy level diagrams and spin-orbit couplings (ξ) between singlet and triplet states for DMACPPY in crystal B; (b) The transition properties of DMACPPY within crystal B.

# References

S1. Maseras, F.; Morokuma, K. IMOMM: A new integrated ab initio + molecular mechanics geometry optimization scheme of equilibrium structures and transition states, *J. Comput. Chem.* 1995, 16, 1170-1179.

S2. Frisch, M.; Trucks, G.; Schlegel, H.; Scuseria, G.; Robb, M.;Cheeseman, J.; Scalmani, G.; Barone, V.; Petersson, G.; Nakatsuji, H. Gaussian 16; Gaussian, Inc.: Wallingford, CT, 2016.

S3. Shao, Y.; Gan, Z.; Epifanovsky, E.; Gilbert, A. T.; Wormit, M.; Kussmann, J.; Lange, A. W.; Behn, A.; Deng, J.; et al. Advances in molecular quantum chemistry contained in the Q-Chem 4 program package, *Mol. Phys.* 2015, 113, 184-215.

S4. Abraham, M. J.; Spoel, D. v. d.; Lindahl, E.; Hess, B., GROMACS User Manual version 5.1. 2015.

S5. Wang, J.; Wolf, R. M.; Caldwell, J. W.; Kollman, P. A.; Case, D. A., Development and Testing of a General Amber Force Field, *J. Comput. Chem.* 2004, 25, 1157-1174.

S6. Bayly, C. I.; Cieplak, P.; Cornell, W.; Kollman, P. A., A well-behaved electrostatic potential based method using charge restraints for deriving atomic charges: the RESP model, *J. Phys. Chem.* 1993, 97, 10269-10280.

S7. Cornell, W. D.; Cieplak, P.; Bayly, C. I.; Kollmann, P. A., Application of RESP Charges to Calculate Conformational Energies, Hydrogen Bond Energies, and Free Energies of Solvation, *J. Am. Chem. Soc.* 1993, 115, 9620-9631.

S8. Bussi, G.; Donadio, D.; Parrinello, M., Canonical sampling through velocity rescaling, *J. Chem. Phys.* 2007, 126, 014101.

S9. Parrinello, M.; Rahman, A., Polymorphic Transitions in Single-Crystals-a-New Molecular-Dynamics Method, *J. Appl. Phys.* 1981, 52, 7182-7190.

S10. Rappe, A. K.; Casewit, C. J.; Colwell, K. S.; Goddard, W. A.; Skiff, W. M., UFF, a full periodic table force field for molecular mechanics and molecular dynamics simulations, *J. Am. Chem. Soc.* 1992, 114, 10024-10035.

S11. Niu, Y. Peng, Q. Shuai, Z. Promoting-mode free formalism for excited state radiationless decay process with Duschinsky rotation effect, *Sci China Ser B-Chem.*, 2008, 51, 1153-1158.

S12. Peng, Q. Yi, Y. Shuai, Z. Shao, J. Toward Quantitative Prediction of Molecular Fluorescence Quantum Efficiency: Role of Duschinsky Rotation, *J. Am. Chem. Soc.* 2007, 129, 9333-9339

S13. Niu, Y. Li, W. Peng, Q. Geng, H. Yi, Y. Wang, L. Nan, G. Wang, D. Shuai, Z. Molecular Materials Property Prediction Package (MOMAP) 1.0: a Software Package for Predicting the Luminescent Properties and Mobility of Organic Functional Materials, *Molecular Physics,* 2018, 116, 1078-1090
